# Traditional birth attendants lack basic information on HIV and safe delivery practices in rural Mysore, India

**DOI:** 10.1186/1471-2458-10-570

**Published:** 2010-09-22

**Authors:** Purnima Madhivanan, Bhavana N Kumar, Paul Adamson, Karl Krupp

**Affiliations:** 1 Public Health Research Institute of India, 89/B, 2nd Cross, 2nd Main, Yadavgiri, Mysore, 570020, India; 2 San Francisco Department of Public Health, 1360 Mission Street, Suite 401, San Francisco, CA 94103, USA

## Abstract

**Background:**

There is little research on HIV awareness and practices of traditional birth attendants (TBA) in India. This study investigated knowledge and attitudes among rural TBA in Karnataka as part of a project examining how traditional birth attendants could be integrated into prevention-of-mother-to-child transmission of HIV (PMTCT) programs in India.

**Methods:**

A cross-sectional survey was conducted between March 2008 and January 2009 among TBA in 144 villages in Mysore *Taluk*, Karnataka. Following informed consent, TBA underwent an interviewer-administered questionnaire in the local language of *Kannada *on practices and knowledge around birthing and HIV/PMTCT.

**Results:**

Of the 417 TBA surveyed, the median age was 52 years and 96% were Hindus. A majority (324, 77.7%) had no formal schooling, 88 (21.1%) had up to 7 years and 5 (1%) had more than 7 yrs of education. Only 51 of the 417 TBA (12%) reported hearing about HIV/AIDS. Of those who had heard about HIV/AIDS, only 36 (72%) correctly reported that the virus could be spread from mother to child; 37 (74%) identified unprotected sex as a mode of transmission; and 26 (51%) correctly said healthy looking people could spread HIV. Just 22 (44%) knew that infected mothers could lower the risk of transmitting the virus to their infants. An overwhelming majority of TBA (401, 96.2%) did not provide antenatal care to their clients. Over half (254, 61%) said they would refer the woman to a hospital if she bled before delivery, and only 53 (13%) felt referral was necessary if excessive bleeding occurred after birth.

**Conclusions:**

Traditional birth attendants will continue to play an important role in maternal child health in India for the foreseeable future. This study demonstrates that a majority of TBA lack basic information about HIV/AIDS and safe delivery practices. Given the ongoing shortage of skilled birth attendance in rural areas, more studies are needed to examine whether TBA should be trained and integrated into PMTCT and maternal child health programs in India.

## Background

Worldwide, it is estimated that there are more than 60 million non-institutional births each year with the vast majority being attended by traditional birth attendants (TBA) [1]. In most developing countries in South Asia, less than half of pregnant women receive trained medical assistance during their delivery[2,3]. While shockingly high, this figure still masks an even greater underlying inequality between urban and rural areas where most of the population resides. In developing countries, rural mothers are 40% less likely to be attended by skilled health personnel compared with their urban counterparts[2].

With almost 60% of births occurring at home[4], India faces a variety of challenges in providing high quality maternal child healthcare. Currently, the estimated maternal mortality ratio (MMR) at 450 per 100,000 live births, casts doubt upon the country's ability to reach its Millennium Development Goal of 109 maternal deaths per 100,000 live births by 2015[1]. The country has also made only slow progress in expanding access to antiretrovirals for prevention of mother-to-child transmission of HIV (PMTCT). Each year only about 20% of the estimated 50,000 HIV positive pregnant women receive prophylaxis to prevent transmission of HIV to their infant [5].

There is growing evidence from Africa and other parts of the world showing that TBA may be able to play an important role in improving maternal child outcomes and preventing HIV. Greenwood *et al*, in their randomized control trial on TBA training in Gambia, demonstrated a 62% reduction in maternal mortality. Their study also showed a significant increase in maternal tetanus immunization rates, and maternal antenatal attendance among mothers delivering with trained TBA[6]. Jokhio and colleagues, in their large cluster randomized intervention study on prevention of perinatal mortality in Pakistan, reported a 20% reduction in perinatal death when TBA were trained and provided with disposable delivery kits[7]. Perez *et al*, in a large feasibility study of TBA willingness to participate in HIV prevention programs in Zimbabwe, found 75% acceptability among TBA to participate in HIV prevention activities such as accompanying new-borns to closest health centre to receive medication (15%) and assisting health centers in documenting ANC-PMTCT services [8]. Another study in Tanzania showed that women receiving HIV advice from a trained and motivated TBA were three times more likely to accept an HIV test and also had a three-fold increased chance of receiving Nevirapine for prevention of mother-to-child transmission of HIV[9].

There are few recent studies on the birthing practices of TBA in India, and none investigating knowledge and attitudes about HIV. Stella Mulder and Karen Trollope-Kumar in studies from different parts of India reported a number of customary practices among TBA. Mulder observed that many attendants were replacing traditional practices for treating the cord stump with ash or dung[10] with more modern remedies such as antiseptic powder. Similarly, Trollope-Kumar observed that many TBA she studied also administered both allopathic and herbal medicines[11]. Other common TBA practices described in these studies included: providing advice on diet and activity[10,11], disposing of the placenta through burial[10,11], delivering women in a supine or crouching position, sprinkling water on the child immediately after birth, and wiping the baby with coconut or castor oil followed by a warm soap bath[10]. Mulder also noted that TBA appeared to have low knowledge regarding many life-threatening complications including excessive bleeding, prolonged labor, and vaginal tears[10].

This study investigated practices and knowledge about birthing and HIV/PMTCT among active TBA attending births in rural areas of Mysore *Taluk *in the south Indian state of Karnataka.

## Methods

### Study Site

Karnataka state with an MMR of 221 per 100,000 live births has the highest rate of maternal death among South Indian States[12]. It has also been identified as one of the 6 high HIV prevalence states in the country. Rates of infection vary substantially across the state's 27 districts with an average of just under 1% of antenatal clinic attendees being infected with HIV[13]. Mysore District is located in the southern part of the state bounded by Mandya district to the northeast, Chamrajanagar district to the southeast, Kerala state to the south, Kodagu district to the west, and Hassan district to the north. The district has an area of 6,854 km^2 ^and a population of 2,641,027, of which about half (49%) are females[14]. Approximately 62.8% of the population live in rural areas. Overall, the literacy rate is 63.5%. Hindus constitute 87.4% of the population, Muslims 8.9%, and Christians, Buddhists and other religious groups about 3.7%. *Kannada *and *Urdu *are the dominant languages in this area.

### Data collection

Between August and November 2009, 511 TBA were screened and 417 enrolled in a descriptive cross-sectional survey. To be eligible, a potential participant had to be able to speak *Kannada *or *Urdu*; understand and give informed consent; and have delivered a minimum of 10 babies in the previous year. The survey was conducted in 144 villages located ten kilometers or more outside of Mysore City since active TBA in Mysore District are typically found in rural areas with poor access to medical care. TBA were identified through key-informant interviews with village elders, community health workers, and recently delivered mothers registered with government health workers. In addition, participating TBA were asked to refer any other birth attendants they knew of in their local area. Three Masters degree-level graduates in Social Work or Psychology were selected as interviewers. All interviewers were trained for four weeks in issues around relationship building, privacy and confidentiality, and methods for administering questionnaires in a standardized, non-judgmental fashion. After undergoing an informed consent process, eligible TBA answered a structured face-to-face interview in *Kannada *in a private setting at their home or place of work. The study was approved by the Vikram Hospital Independent Ethics Committee.

### Statistical analysis

Data were analyzed using Stata 10.1 (Stata Corporation, College Station, TX). Descriptive statistical methods were used to provide a general profile of the study population. Analyses were conducted using Pearson chi-squared or Fisher-exact tests for categorical variables and *t-tests *for comparison of continuous variables.

## Results

### Demographics

Of the 511 TBA screened, 428 (83.7%) were found eligible for the study. Reasons for non-eligibility included having delivered less than 10 babies in the previous year or being unable to give informed consent. Of the 428 eligible women, 417 (97.4%) agreed to be interviewed. The median age of study participants was 52 years (range: 26-80 years) with 77 (18%) aged 40 years or less, 138 (33%) between 41-54 years, and 202 (48%) over 55 years of age. A large majority (324, 77.7%) had no formal education, 88 (21%) had between one and seven years and five (1.2%) had more than seven years of education. Among these participants, 415 (99.5%) were Hindus and two (0.48%) were Muslim. None of the participants reported full time employment as a TBA.

### Knowledge, Attitudes and Beliefs about HIV/AIDS

Only 51 of the 417 (12.3%) TBA reported hearing about HIV/AIDS. Among those, 25 (49%) wrongly believed that only 'sick looking' people could spread infection, and 21 (42%) thought HIV could be transmitted through touching or hugging. Additionally, 32 (64%) reported that HIV could be spread by mosquitoes and 11 (21.6%) were not aware that the virus could be transmitted through blood or blood products. When asked about vertical transmission of HIV, 14 (28%) did not know that HIV could be spread from mother to child and 28 (56%) were unaware that infected mothers could deliver healthy infants. When TBA were asked what precautions they took to protect themselves against HIV, 39 (76%) said they avoided attending births for women they suspected were HIV-infected, 3 (6%) reported wearing rubber gloves during deliveries, 2 (4%) said they rubbed oil on their hands prior to carrying out procedures, 3 (6%) reported taking herbal remedies before deliveries, 2 (4%) cleaned all surfaces after each procedure, and 1 (2%) reported using sterilized blades and instruments.

### TBA Birthing Practices

#### Antepartum Practices

A large majority (401, 96.2%) of TBA reported not providing antenatal care for the woman prior to the onset of labor contractions. About 372 TBA (89%) advised women to use special postures when seated, 354 (85%) advised special postures for sleep, 282 (68%) prescribed special foods and diets, 255 (61%) told women to massage their abdomen with special oils, and 182 (43%) suggested exercise. When participants were asked how abortions were carried out in their village, 88 (21%) reported consumption of raw papaya, 61 (14.6%) reported consumption of heat producing foods, and 318 (76%) reported they didn't know.

#### Intrapartum Practices

Almost all TBA (404, 96.9%) reported telling their clients to contact them only at the onset of fast painful contractions. Among the remainder, 23 (6%) advised waiting until the water breaks, and 10 (2.4%) told their clients to notify them once the child's head crowned. The supine position was the most commonly reported delivery posture (228, 54.7%), followed by squatting (140, 33.6%), sitting (105, 25.2%) and kneeling (43, 10.3%). When asked what tools were used during deliveries, almost all (98%) reported using a blade, 25 (6%) a scissors, 2 (0.5%) forceps, and 2 (0.5%) a suction bulb. Just over half (183, 51.9%) reported sterilizing their equipment prior to a delivery. When TBA were asked whether they would ever refer women to a medical center, 70% said they would if the baby was stuck, if a mother had excessive bleeding before delivery (60.9%) and if the baby came out the wrong way (56.8%). Other reasons reported for hospital referrals were: if the umbilical cord came out before the baby (37.9%), if there was excessive bleeding following birth (12.7%), if the placenta was stuck (11.2%), and if the umbilical cord was wrapped around the baby's head (4.6%) (Table 1).

**Table 1:** Birthing practices of traditional birth attendants in Mysore *Taluk*, India (N = 417)

Characteristic	Total	
	**N**	**(%)**
Would you consider this a high-risk pregnancy		
Age <17 & >35 yrs	210	50.4
First pregnancy or >5 times pregnant	163	39.1
Past h/o spontaneous abortion	208	49.9
Past h/o blood pressure	245	58.8
Past h/o caesarian section	267	64.0
Past h/o postpartum hemorrhage	178	42.7
Stillbirth during last delivery	228	54.7
Abnormal presentation of fetus	288	69.1
Mother's height <1.45 m	171	41.0
Fetus stops moving or unable to hear the baby	288	69.1
Twins	228	54.7
Do you advise any of the following treatments		
Special food and diet	282	67.6
Enema	13	3.1
Exercise	182	43.6
Massage with oil on stomach	255	61.2
Special posture to sit	372	89.2
Special posture to sleep	354	84.9
Medicines	78	18.7
Pushing out the abdomen during the pain	191	45.8
When do you advise your clients to get you when they are ready to deliver		
Breaking of bag of waters	23	5.5
Fast painful contractions	404	96.9
Head is coming out	10	2.4
What is the most common posture for delivering a woman in labor		
Seated	105	25.2
Squatting	140	33.6
Laying down	228	54.7
Kneeling	43	10.3
What would you do if there is excessive bleeding **before **delivery		
Refer to medical center	254	60.9
Do nothing, will have normal delivery	6	1.44
Don't know	19	4.56
What would you do if there is excessive bleeding **after **delivery		
Wait until 'bad blood' has passed	414	99.3
Refer to medical center	53	12.7
What would you do: After the baby is delivered, the placenta becomes stuck inside the woman or it does not come out right away		
Massage the uterus	134	32.1
Manually clear the placenta by hand	50	11.9
Induce vomiting by stuffing hair into mouth in order to expel placenta	65	15.6
Induce vomiting by sticking hand into mouth in order to expel placenta	18	4.3
Drink castor oil	1	0.2
Refer to medical center	47	11.2
Don't know	11	2.6
What would you do if:		
Umbilical cord comes out of the woman before the baby		
Deliver baby normally	15	3.6
Manually push the cord back inside the woman	54	12.9
Rotate the baby's position inside the mother	11	2.6
Refer to medical center	158	37.9
Don't know	156	37.4
Refuse to answer	15	3.6
Umbilical cord is wrapped around the baby's head		
Leave the cord until the baby is delivered	104	24.9
Loosen the cord around the baby's neck	239	57.3
Tie and cut the cord after the shoulders come through the pelvis	15	3.6
Refer to medical center	19	4.6
Don't know	15	3.6
Refuse to answer	3	0.72
Baby is stuck inside the mother		
Change the position of the mother	13	3.1
Refer to the medical center	292	70.0
Don't know	12	2.88
Refused to answer	5	1.2
Baby comes out the wrong way		
Deliver the baby normally	123	29.5
Refer to medical center	237	56.8
Don't know	39	9.35
Refuse to answer	3	0.72
Mother gets a fever, feels dizzy or becomes pale		
Give mother water to drink	92	22.1
Refer to medical center	194	46.5
Don't know	38	9.1
Refuse to answer	1	0.24
There are cuts and tears in the woman's vaginal wall after birth		
Wash with soap and water	93	22.3
Apply turmeric with oil	11	2.6
Don't know	133	31.9
Refused to answer	9	2.2
A newborn does not cry		
Blow in the baby's ear	115	27.6
Splash baby with cold water	126	32.2
Massage baby's back, feet or hands	108	25.9
Hold baby with legs upward	65	15.6
Flick the baby's hands or feet	82	19.7
Refer to medical center	22	5.3

#### Postpartum Practices

If the baby did not cry immediately after delivery, 126 (32.6%) TBA reported splashing the baby with cold water; 115 (27.6%) blew in the baby's ear; 108 (25.9%) massaged the baby's back, hands and feet; 82 (19.7%) flicked the baby's feet, 65 (15.6%) held the baby upside down and 22 (5.3%) would refer the mother and infant to a medical center. Less than a third of participants (113, 27.1%) said they advised clients to breastfeed immediately after delivery. About half, 186 (44.6%) told women that they could breastfeed after several hours, while 99 (23.7%) advised waiting for 24 hours and 19 (4.6%) specified other periods. Most (77.7%) reported telling clients that colostrum was good for the baby, but 91 (21.8%) advised against it.

## Discussion

This study shows that knowledge about HIV/AIDS is extremely low among TBA living in rural areas of Mysore *Taluk*. An overwhelming majority (88%) of surveyed birth attendants had not even heard of HIV/AIDS, a surprising finding given that the epidemic in India is more than two decades old. This is in stark contrast to high levels of knowledge found by the government of India among the general population in HIV sentinel surveillance surveys. The 2006 National Behavioral Surveillance Survey showed 75 percent of people living in rural areas were aware of HIV/AIDS[15]. Even the small number of TBA in our study, who had knowledge about HIV demonstrated poor understanding about how the virus was transmitted. Additionally, less than half of those were aware of the potential for preventing vertical transmission of HIV. Finally, 39 of the 51 TBA (76%) who had said they had heard of HIV/AIDS, expressed unwillingness to attend the births of HIV-infected mothers.

The level of knowledge about safe birthing practices was also low among TBA. Considering that obstetrical hemorrhage and sepsis are still the leading cause of maternal death in India[16], it is not surprising that the study found only 13% of TBA referred mothers experiencing excessive bleeding following birth to a medical center, and that only about half (51.9%) sterilized equipment prior to deliveries. Other 'unsafe' procedures still practiced among TBA included sucking secretions out of a baby's mouth and nose with their mouth, applying cow dung, *ghee *(clarified butter) and other preparations on the umbilical cord, and inducing vomiting by stuffing hair in a woman's throat to stimulate contractions of the uterus to clear the placenta. Even more concerning, TBA appeared to have low levels of awareness about when clients should be referred to a hospital. A small number of TBA (4.6%) said they would refer a mother to a medical center if the umbilical cord was wrapped around the baby's head, and just over half (56.8%) would refer the mother if the baby was coming out the wrong way. Most TBA but not all (70%) would refer the woman if the baby was stuck inside the birth canal.

While knowledge deficits on both HIV and birthing practices among TBA appear large, they underline a continuing dilemma for countries like India where more than 60% of the population lives in rural areas[17]. While the Government of India has made substantial investments in increasing rural healthcare through its National Rural Health Mission, access to skilled birth attendance is still an unrealized goal for much of the population. Integrating TBA in the rural health system may be a viable short-term solution for lowering maternal and infant mortality in rural areas with limited access to medical care. Additionally, as some studies in Africa have shown[8,18], TBA could contribute to HIV prevention efforts, particularly those focused on mother-to-child transmission of HIV. Studies in India have already demonstrated that training TBA can improve newborn care[19], increase uptake of maternal health services[20], and reduce neonatal mortality[21]. The difficulties of training TBA should not be underestimated however. Many have low levels of literacy and some may not be interested in further training. Attention must be given to making instruction relevant to the local cultural milieu in which TBA practice and germane to the realities of rural birth attendance. Furthermore, any effort to integrate TBA must be harmonized with national health policies and the existing priorities of the National Rural Health Mission. Given all these challenges, training TBA may still be India's best hope for reaching Millennium Development Goal 5 by 2015.

This study has several limitations. First, it was carried out among rural TBA living in Mysore *Taluk *and may not be representative of TBA in other areas of India. In addition, it was impossible to verify how large a practice each TBA had since this was self reported and might have been subject to recall and information bias. Second, respondents' accounts of events around pregnancy and childbirth may be subject to recall and information bias. It is likely that the TBA may have underreported some practices leading to a conservative estimate of our findings because of social desirability bias. Finally, it is likely that some of the questions were not answered by the respondents, as they may have not understood the vocabulary or terminology in spite of the fact that the questions were from formative research conducted prior to this study among TBA in the region.

## Conclusion

Traditional birth attendants will continue to play an important role in maternal child health in India for the foreseeable future. This study demonstrates that a majority of TBA lack basic information about HIV/AIDS and safe delivery practices. Given the ongoing shortage of skilled birth attendance in rural areas, more studies are needed to examine whether TBA should be trained and integrated into PMTCT and maternal child health programs in India.

## List of Abbreviations

AIDS: Acquired Immuno Deficiency Syndrome; HIV: Human Immunodeficiency Virus; MMR: Maternal Mortality Ratio; PMTCT: Prevention of Mother to Child Transmission of HIV; TBA: Traditional Birth Attendants; UNICEF: United Nations Children's Fund; WHO: World Health Organization.

## Competing interests

The authors declare that they have no competing interests.

## Authors' contributions

PM and KK were involved in the conception and design of the study. BNK and PA were responsible for acquisition of data and PM analyzed the data. PM and KK drafted the article and all authors (PM, BNK, PA, KK) participated in interpreting the data and critically revising the manuscript for important intellectual content. All authors read and approved the final version of the manuscript to be published.

## Pre-publication history

The pre-publication history for this paper can be accessed here:

http://www.biomedcentral.com/1471-2458/10/570/prepub

## References

[B1] UNICEF State of the World's Children 2009: Maternal and Newborn Health. New York 2009

[B2] Childinfo: Monitoring the situation of women and children http://www.childinfo.org/delivery_care.html

[B3] McClure EM Wright LL Goldenberg RL Goudar SS Parida SN Jehan I Tshefu A Chomba E Althabe F Garces A The global network: a prospective study of stillbirths in developing countries Am J Obstet Gynecol 2007 197 3 247 e241-245 10.1016/j.ajog.2007.07.004 17826406 PMC2150563

[B4] Mavalankar D Vora K Prakasamma M Achieving Millennium Development Goal 5: is India serious? Bulletin of the World Health Organization 2008 86 4 243 243A 10.2471/BLT.07.048454 18438507 PMC2647422

[B5] WHO PMTCT strategic vision 2010-2015: preventing mother-to-child transmission of HIV to reach the UNGASS and Millennium Development Goals 2010 Geneva: World Health Organization

[B6] Greenwood AM Bradley AK Byass P Greenwood BM Snow RW Bennett S Hatib-N'Jie AB Evaluation of a primary health care programme in The Gambia. I. The impact of trained traditional birth attendants on the outcome of pregnancy The Journal of tropical medicine and hygiene 1990 93 1 58 66 2304134

[B7] Jokhio AH Winter HR Cheng KK An intervention involving traditional birth attendants and perinatal and maternal mortality in Pakistan The New England journal of medicine 2005 352 20 2091 2099 10.1056/NEJMsa042830 15901862

[B8] Perez F Aung KD Ndoro T Engelsmann B Dabis F Participation of traditional birth attendants in prevention of mother-to-child transmission of HIV services in two rural districts in Zimbabwe: a feasibility study BMC public health 2008 8 401 10.1186/1471-2458-8-401 19061506 PMC2612666

[B9] Msaky H Kironde S Shuma J Nzima M Mlay V Reeler A Scaling the frontier: traditional birth attendant involvement in PMTCT service delivery in Hai and Kilombero districts of Tanzania. abstract no.: ThPeE8084 International Conference on AIDS 2004; Bangkok, Thailand 2004

[B10] Mulder S Midwifery in rural India: a study of traditional birth attendants in Tamil Nadu, India Aust Coll Midwives Inc J 1995 8 1 24 30 10.1016/S1031-170X(05)80009-1 7619014

[B11] Trollope-Kumar K The Traditional Birth Attendant in Garhwal, India: Towards a Culturally Relevant Training Program Canadian Journal of Midwifery Research and Practice 2002

[B12] Special Bulletin on Maternal Mortality in India 2004-06 http://censusindia.gov.in/Vital_Statistics/SRS_Bulletins/MMR-Bulletin-April-2009.pdf

[B13] Karnataka State Profile http://newdelhi.usembassy.gov/pepfars/pepfarstateskar.pdf

[B14] Mysore District http://www.mysore.nic.in/index.htm

[B15] National AIDS Control Organisation National Behavioural Surveillance Survey (BSS) 2006 2006 http://www.nacoonline.org/upload/NACO%20PDF/General_Population.pdf

[B16] Kausar R Maternal Mortality in India - Magnitude, Causes and Concerns Indian Journal for the Practising Doctor 2005 2 2

[B17] India: Urban Poverty Report 2009: Factsheet http://data.undp.org.in/poverty_reduction/Factsheet_IUPR_09a.pdf

[B18] Wanyu B Diom E Mitchell P Tih PM Meyer DJ Birth attendants trained in "Prevention of Mother-To-Child HIV Transmission" provide care in rural Cameroon, Africa Journal of midwifery & women's health 2007 52 4 334 341 10.1016/j.jmwh.2006.12.018 17603955

[B19] Satishchandra DM Naik VA Wantamutte AS Mallapur MD Impact of training of traditional birth attendants on the newborn care Indian J Pediatr 2009 76 1 33 36 10.1007/s12098-008-0229-9 19127353

[B20] Mathur HN Sharma PN Jain TP The impact of training traditional birth attendants on the utilisation of maternal health services J Epidemiol Community Health 1979 33 2 142 144 10.1136/jech.33.2.142 490094 PMC1051939

[B21] Bang AT Bang RA Baitule SB Reddy MH Deshmukh MD Effect of home-based neonatal care and management of sepsis on neonatal mortality: field trial in rural India Lancet 1999 354 9194 1955 1961 10.1016/S0140-6736(99)03046-9 10622298

